# Supplementary material for: Ultra-processed food intake in association with BMI change and risk of overweight and obesity: A prospective analysis of the French NutriNet-Santé cohort
Source: PLoS Med. 2020 Aug 27;17(8):e1003256. doi: 10.1371/journal.pmed.1003256 (PMC7451582; doi:10.1371/journal.pmed.1003256)
Supplement: S1 Appendix — (DOCX) [file pmed.1003256.s002.docx]

Online Supplementary Material

**Ultra-processed food intake in association with**

**BMI change and risk of overweight and obesity;**

**a *prospective analysis of the French NutriNet-Santé cohort***

Marie Beslay^∞1^, Bernard Srour^∞^*^1^, Caroline Méjean^2^, Benjamin Allès^1^, Thibault Fiolet^1^, Charlotte Debras^1^, Eloi Chazelas^1^, Mélanie Deschasaux^1^, Méyomo Gaelle Wendeu-Foyet^1^, Serge Hercberg^1,3^, Pilar Galan^1^, Carlos A. Monteiro^4^, Valérie Deschamps^5^, Giovanna Calixto Andrade^1,6^, Emmanuelle Kesse-Guyot^1^, Chantal Julia^≠1,3^, Mathilde Touvier^≠1^

**^∞^ *and ^≠^: equal contributions***

[*b.srour@eren.smbh.univ-paris13.fr](mailto:*b.srour@eren.smbh.univ-paris13.fr)

**Method A:**

**Identification procedure of energy under-reporting in the NutriNet-Santé cohort**

Energy underreporting was identified using Black’s method [1,2] based on the original method developed by Goldberg et al [3], relying on the hypothesis that energy expenditure and intake, when weight is stable, are equal. Black’s equations are based on an estimate of the person’s basal metabolic rate (BMR) calculated via Schofield’s equations [4] and taking into account sex, age, height and weight, as well as physical activity level (PAL), number of 24h records, intra-individual variabilities of reported energy intake and BMR, and intra/inter-variabilities of PAL. In the present study, intra-individual coefficients of variations for BMR and PAL were fixed using the values proposed by Black et al., i.e. 8.5 % and 15%, respectively. For identifying under-reporters, the 1.55 value of PAL was used. It corresponds to the WHO value for “light” activity, which is the probable minimum energy requirement for a normally active but sedentary individual (not sick, disabled or frail elderly). A higher value might have exaggerated the extent of under-reporting. Some under-reporting individuals were not excluded if their reported energy intake, initially estimated abnormally low, was found to be likely in case of recent weight variation or reported practice of weight-loss restrictive diet or proactive statement of the participant that he/she ate less than usual on the day of the dietary record. In the cohort, 20.0 % of the subjects were considered as under-reporters and were excluded from the analyses.

**Method B:**

**Definitions, precisions and examples of ultra-processed foods according to the NOVA classification**

All food and beverage items of the NutriNet-Santé composition table (n=3500) were categorized by a team of three trained dieticians into one of the four NOVA groups, a food classification system based on the extent and purpose of industrial food processing [5–7]. The whole classification was then reviewed by a committee composed of the three dietitians and five researchers, specialists in nutritional epidemiology. In case of uncertainty for a given food/beverage item, a consensus was reached among researchers based on the percentage of home-made and artisanal foods versus industrial brands reported by the participants. The “ultra-processed foods” group of the NOVA classification is the primarily focus of this study. Products in this group undergo industrial processes that include for instance hydrogenation, hydrolysis, extruding, moulding, reshaping, and pre-processing by frying. Flavouring agents, colours, emulsifiers, humectants, non-sugar sweeteners and other cosmetic additives are often added to these products to imitate sensorial properties of unprocessed or minimally processed foods and their culinary preparations. The UPF group is defined by opposition to the other NOVA groups: “unprocessed or minimally processed foods” (fresh, dried, grounded, chilled, frozen, pasteurized or fermented staple foods such as fruits, vegetables, pulses, rice, pasta, eggs, meat, fish or milk), “processed culinary ingredients” (salt, vegetable oils, butter, sugar and other substances extracted from foods and used in kitchens to transform unprocessed or minimally processed foods into culinary preparations) and “processed foods” (canned vegetables with added salt, sugar-coated dry fruits, meat products only preserved by salting, cheeses and freshly made unpackaged breads, and other products manufactured with the addition of salt, sugar or other substances of the “processed culinary ingredients” group). As previously described[8], home-made and artisanal food preparations were identified and decomposed using standardized recipes, and the NOVA classification was applied to their ingredients. Examples of such products as well as examples of distinctions between ultra-processed products and products from other NOVA categories are provided below:

Examples of typical ultra-processed foods according to the NOVA classification:

*Poultry and fish nuggets and sticks and other reconstituted meat products transformed with addition of preservatives other than salt (e.g nitrites); instant noodles and dehydrated soups; carbonated diet and regular sodas; chocolate with emulsifiers, chewing gums and candies with dyes (confectionery); margarine; instant desserts; most breakfast ‘cereals’, ‘energy’ bars; ‘energy’ drinks; flavoured milk drinks; sweet desserts made from fruit with added sugars, artificial flavours and texturizing agents; cooked seasoned vegetables with ready-made sauces; vegetable patties (meat substitutes) containing food additives; ‘health’ and ‘slimming’ products such as powdered or ‘fortified’ meal and dish substitutes.*

For instance, salted-only red or white meats are considered as “processed foods” whereas smoked or cured meats with added nitrites and conservatives, such as sausages and ham are classified as “ultra-processed foods”.

Similarly, canned salted vegetables are considered as “processed foods” whereas industrial cooked or fried seasoned vegetables, marinated in industrial sauces with added flavourings are considered as “ultra-processed foods”.

Flavoured breakfast cereals with added emulsifiers, texturizing agents and/or colorants were included in the ultra-processed food group. Homemade granola, oatmeal, rye and barley flakes without additives were not considered as ultra-processed.

Regarding soups, canned liquid soups with added salts, herbs and spices are considered as “processed foods” while instant dry soup mixes are considered as “ultra-processed foods”.

Example of list of ingredients for an industrial Chicken and Leek flavour soup considered as “ultra-processed” according to the NOVA classification: *“Dried Glucose Syrup, Potato Starch, Flavourings, Salt, Leek Powder (3.6%), Dried Leek (3.5%), Onion Powder, Dried Carrot, Palm Oil, Dried Chicken (0.7%), Garlic Powder, Dried Parsley, Colour [Curcumin (contains MILK)], Ground Black Pepper, MILK Protein, Stabilisers (Dipotassium Phosphate, Trisodium Citrate)”*.

**Examples of food products considered as ultra-processed according to the NOVA classification**

| **Ultra-processed food group** | **Examples of foods** |
| --- | --- |
| Beverages | Sugary drinks (e.g. regular sodas, sugary fruit-based and flavoured beverages, industrial chocolate powder beverages, energy drinks, flavoured waters); artificially sweetened beverages (e.g. diet sodas, artificially sweetened ice teas) |
| Dairy products | Flavoured or artificially sweetened yoghurts; products such as dairy desserts, cream cheese, milkshakes, dairy beverages, flavoured milk with one or more texturizer, emulsifier, colorant or other cosmetic additives |
| Fats and sauces | Sauces and dressings (salad dressing, mayonnaise, ketchup, béchamel, and other dressings) containing emulsifiers, texturizers, flavour enhancers or other additives |
| Fruits and vegetables | Instant powder soups; reconstituted vegetarian/soy steaks with additives; flavoured and artificially sweetened fruit compotes; vegan nuggets |
| Meat, fish, and eggs | Processed meat with added nitrites; chicken nuggets; fish fingers; industrial ‘cordon bleu’ chicken with wheat dextrose, emulsifiers, preservatives; surimi-crab sticks |
| Starchy foods and cereals | Flavoured breakfast cereals with added emulsifiers, texturizing agents and/or colorants; industrial pre-baked breads and buns with added dextrose, preservatives or emulsifiers. |
| Sugary products | Industrially packed cookies, cakes, chocolate/wafer bars, and candies manufactured with glucose syrup, modified starch, hydrogenated oils, colours, flavours, emulsifiers. |
| Salty snacks | Chips, crisps and crackers made with other ingredients than potatoes, oil and salt such as maltodextrin, flavors, dyes, emulsifiers, flavour enhancers |

**Figure A: Density plot of the distribution of the proportion of UPF in the diet, NutriNet-Santé, 2009-2019 (N=110,260)**


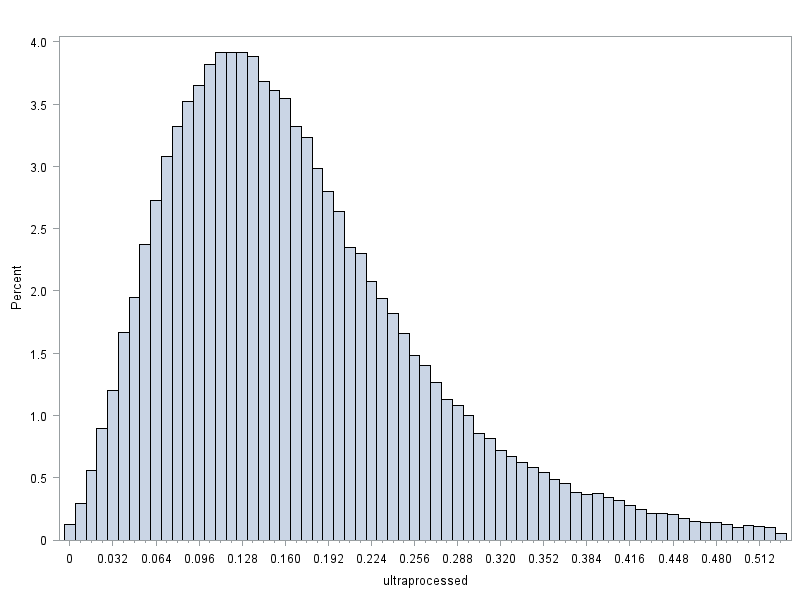


**Method C:**

**Computation of IPAQ physical activity levels** [9,10]

The description of physical activity and inactivity was performed using a questionnaire implemented on the Internet, the International Physical Activity Questionnaire (IPAQ). This questionnaire allows categorization of physical activity, according to three levels of intensity (walking, medium intensity activities and high intensity activities), in terms of weekly frequency (number of days / week) and daily duration (in minutes) corresponding to each level. Thanks to the information gathered in these three categories of energy expenditure, it is possible to estimate, by following the rules recommended by the IPAQ research committee, a weekly energy expenditure in Metabolic Equivalents, expressed in MET-minutes / week.

|  | Number of days/week | Daily duration (minutes) | Metabolic Equivalents (MET-minutes/week) |
| --- | --- | --- | --- |
| High intensity activities | J1 | D1 | MET1 = 8.0 x J1 x D1 |
| Medium intensity activities | J2 | D2 | MET2 = 4.0 x J2 x D2 |
| Walking | J3 | D3 | MET3 = 3.3 x J3 x D3 |

Participants can therefore be classified into one of the three categories: High physical activity level, Moderate and Low. The categorization is performed as follows:

| Physical activity level | Algorithm of categorization |
| --- | --- |
| High physical activity level | - J1 ≥ 3 and MET1 ≥ 1 500 or - J1 + J2+ J3 ≥ 7 and MET1 + MET2 + MET3 ≥ 3 000 |
| Moderate physical activity level | - J1 ≥ 3 and D1 ≥ 20 or - J2 ≥ 5 and D2 ≥ 30 or J3 ≥ 5 and D3 ≥ 30 or - J1 + J2+ J3 ≥ 5 and MET1 + MET2 + MET3 ≥ 600 |
| Low physical activity level | - No reported physical activity - None of the above-mentioned criteria is matched |

**Method D:** **Method for deriving dietary patterns by principal component analysis and corresponding factor loadings**

Dietary patterns were produced from principal-components analysis based on 20 predefined food groups, using the SAS ‘‘Proc Factor’’ procedure (SAS Institute Inc., Cary, North Carolina). This factor analysis forms linear combinations of the original food groups, thereby grouping together correlated variables. Coefficients defining these linear combinations are called factor loadings. A positive factor loading means that the food group is positively associated with the factor, whereas a negative loading reflects an inverse association with the factor. For interpreting the data, we considered foods with a loading coefficient under -0.25 or over 0.25. We rotated factors by orthogonal transformation using the SAS ‘‘Varimax’’ option to maximize the independence (orthogonality) of retained factors and obtain a simpler structure for easier interpretation. In determining the number of factors to retain, we considered eigenvalues greater than 1.25, the scree test (with values being retained at the break point between components with large eigenvalues and those with small eigenvalues on the scree plot), and the interpretability of the factors. For each subject, we calculated the factor score for each pattern by summing observed consumption from all food groups, weighted by the food group factor loadings. The factor score measures the conformity of an individual’s diet to the given pattern. Labeling was descriptive, based on foods most strongly associated with the dietary patterns. The healthy pattern (explaining 10.6% of the variance) was characterized by higher intakes of fruit, vegetables, soups and broths, unsweetened soft drinks and whole grains and lower sweetened soft drinks intake. The Western pattern (explaining 7.0% of the variance) was characterized by higher intakes of fat and sauces, alcohol, meat and starchy foods.

|  | Factor loadings | |
| --- | --- | --- |
|  | Healthy Pattern | Western Pattern |
| Alcoholic drinks | -.09 | 0.28 |
| Breakfast cereals | 0.07 | -.18 |
| Cakes and biscuits | -.19 | 0.00 |
| Dairy products | 0.06 | -.014 |
| Eggs | 0.07 | 0.04 |
| Fats and sauces | 0.01 | 0.54 |
| Fish and seafood | 0.20 | 0.10 |
| Fruit | 0.35 | 0.05 |
| Meat | -.18 | 0.32 |
| Pasta and rice | -.21 | 0.34 |
| Potatoes and tubers | -.03 | 0.40 |
| Poultry | -.03 | 0.06 |
| Processed meat | -.23 | 0.21 |
| Pulses | 0.19 | 0.03 |
| Soups and broths | 0.26 | 0.23 |
| Sugar and confectionery | -.09 | 0.12 |
| Sweetened soft drinks | -.29 | -.00 |
| Unsweetened soft drinks | 0.26 | 0.15 |
| Vegetables | 0.47 | 0.23 |
| Whole grains | 0.38 | -.04 |

**Figure B: Cox models assumption testing:**

Results of proportional risk assumption using Schoenfeld residuals (figures B1)

***Overweight***


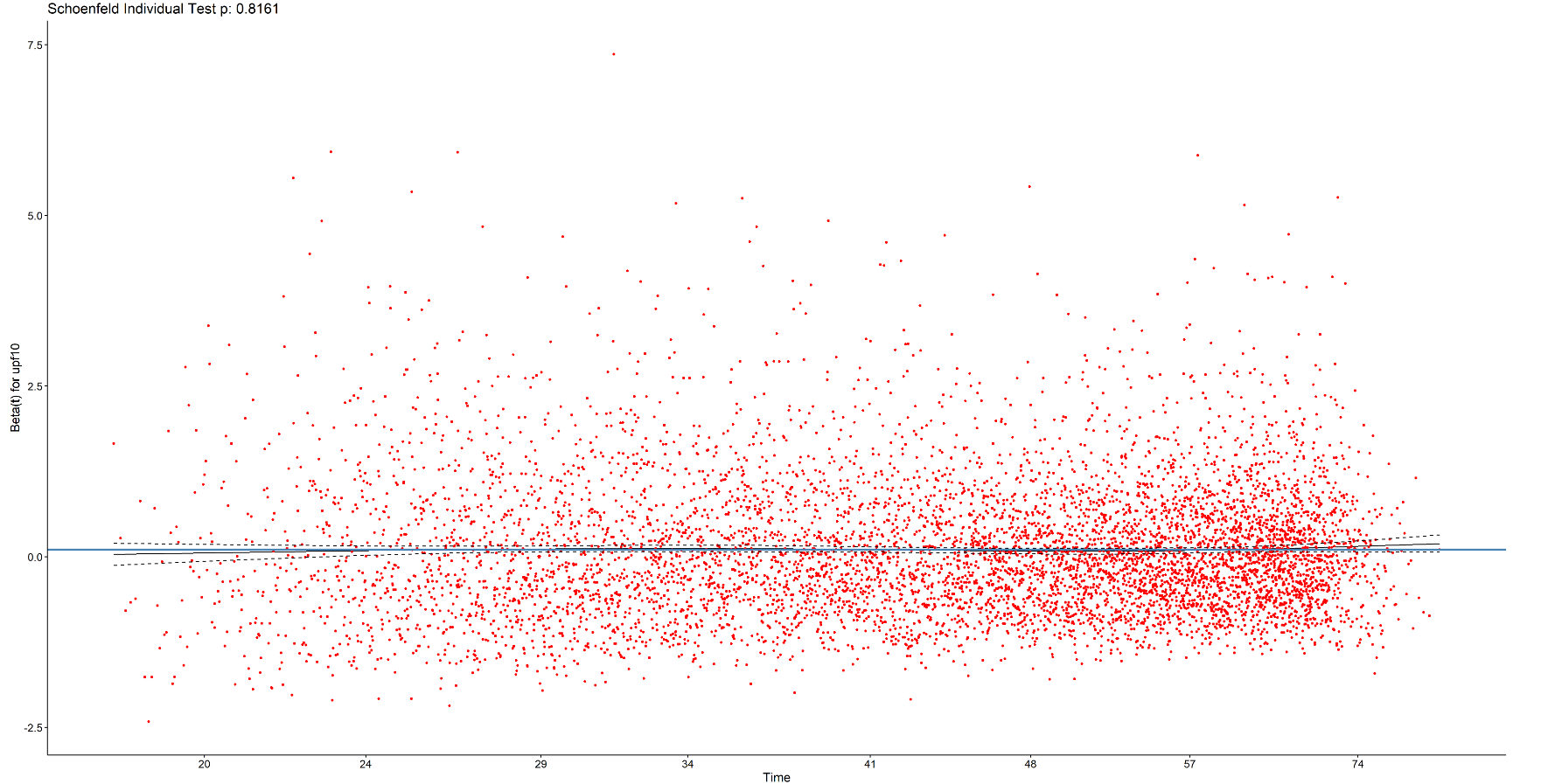


***Obesity***


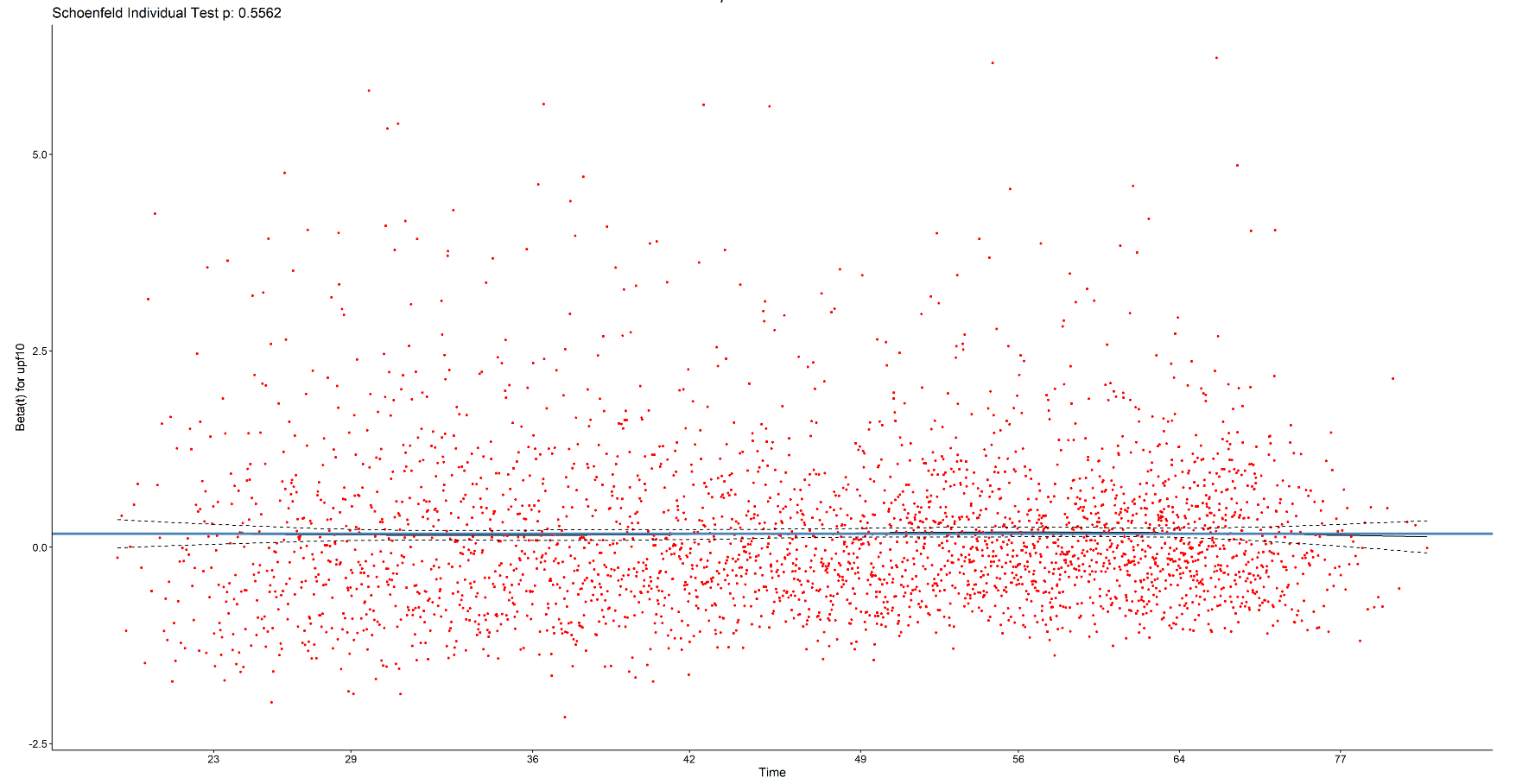


Results of linearity assumption using Martingale residuals (figures B2)

***Overweight***

***
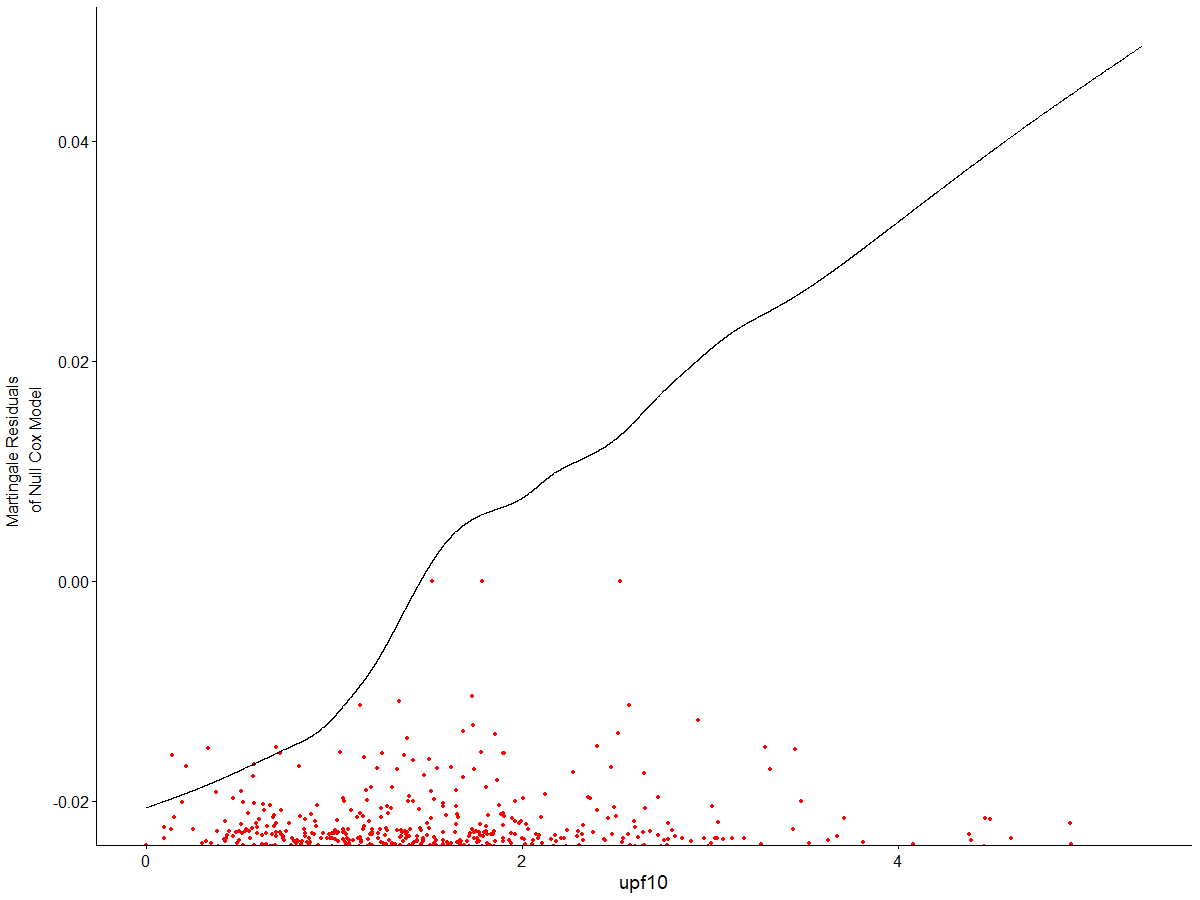
***

***Obesity***

***
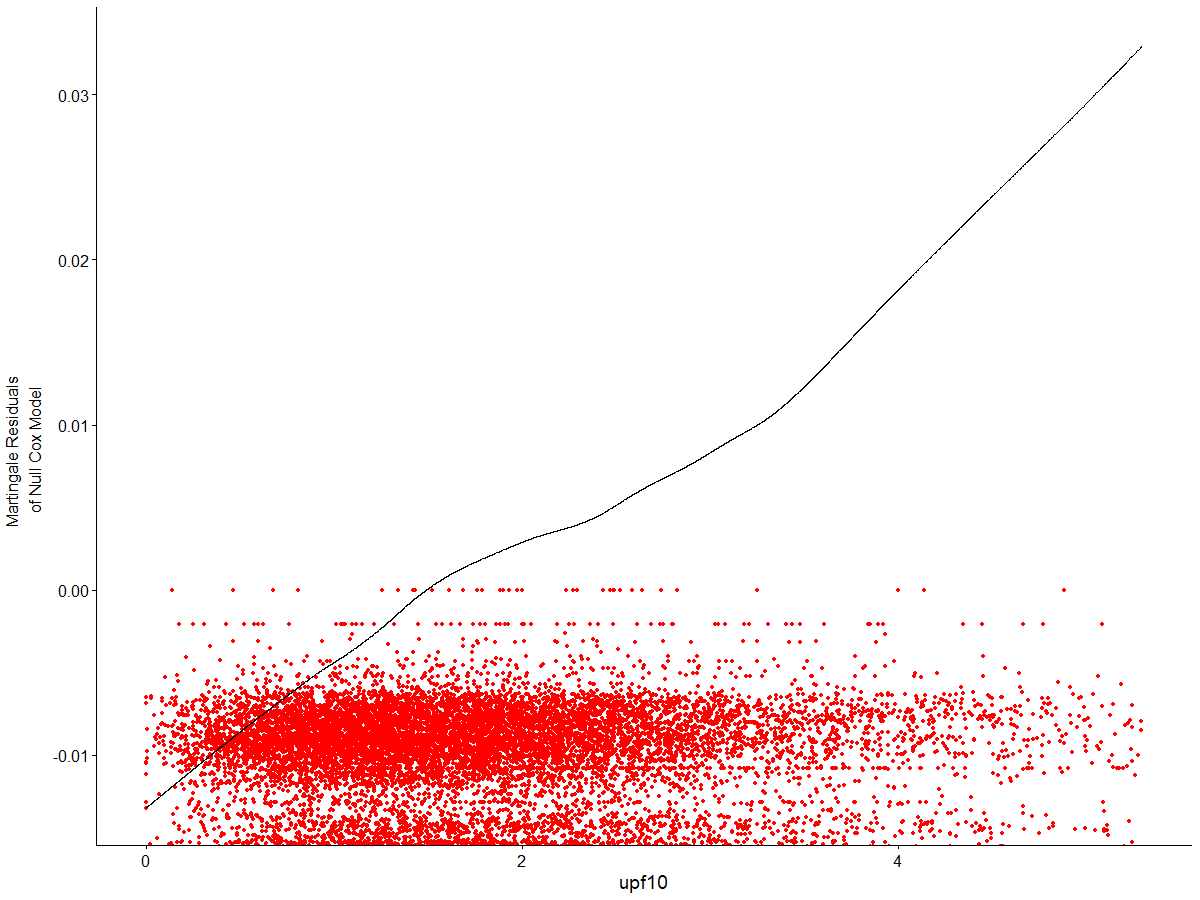
***

**Table A** – Sensitivity analyses: Associations between ultra-processed food (UPF) intake and risks of overweight and obesity from multi-adjusted Cox proportional hazard models after sensitivity and stratified analyses, NutriNet-Santé cohort, France, 2009 – 2019^a^

|  | **Overweight risk** | | | **Obesity risk** | | |
| --- | --- | --- | --- | --- | --- | --- |
|  | **N cases/non-cases** | **HR^*^ (95% CI)** | **P-value** | **N cases/non-cases** | **HR^*^ (95% CI)** | **P-value** |
| After excluding cases of the first two years of follow-up | 3397 / 47974 | 1.12 (1.08 to 1.16) | <0.001 | 1543 / 68805 | 1.13 (1.07 to 1.18) | <0.001 |
| UPF proportion in the diet weighted by energy | 7063 / 47974 | 1.06 (1.04 to 1.08) | <0.001 | 3066 / 68805 | 1.03 (1.00 to 1.05) | 0.03 |
| Further adjustment for time spent sitting down or being sedentary | 6440 / 44040 | 1.10 (1.08 to 1.13) | <0.001 | 2759 / 63096 | 1.08 (1.04 to 1.12) | <0.001 |
| Multiple imputation using MICE^b^ | 7063 / 47974 | 1.11 (1.08 to 1.13) | <0.001 | 3066 / 68805 | 1.08 (1.05 to 1.12) | <0.001 |
| Complete case analysis^c^ | 5664 / 39360 | 1.10 (1.07 to 1.14) | <0.001 | 2460 / 56261 | 1.10 (1.06 to 1.14) | <0.001 |
| In men | 1612 / 8494 | 1.08 (1.02 to 1.13) | 0.003 | 638 /15228 | 1.02 (0.94 to 1.10) | 0.5 |
| In women | 5451 / 39480 | 1.12 (1.09 to 1.15) | <0.001 | 2428 / 53577 | 1.10 (1.06 to 1.14) | <0.001 |
| In younger adults (<=45 years old) | 3689 / 27004 | 1.13 (1.09 to 1.16) | <0.001 | 1441 / 35488 | 1.09 (1.03 to 1.14) | <0.001 |
| In older adults (> 45 years old) | 3374 / 20970 | 1.07 (1.02 to 1.11) | <0.001 | 1625 / 33317 | 1.09 (1.03 to 1.14) | 0.001 |
| In participants having lower sugar intakes  (≤ median=89.9 g) | 3693 / 23825 | 1.07 (1.03 to 1.11) | <0.001 | 1735 / 34201 | 1.07 (1.02 to 1.13) | 0.002 |
| In participants having higher sugar intakes  (> median=89.9 g) | 3370 / 24149 | 1.14 (1.11 to 1.18) | <0.001 | 1331 / 34604 | 1.09 (1.04 to 1.15) | <0.001 |
| In participants having lower SFA intakes  (≤ median=31.2 g) | 3405 / 24114 | 1.10 (1.07 to 1.14) | <0.001 | 1536 / 34400 | 1.06 (1.01 to 1.12) | 0.007 |
| In participants having higher SFA intakes  (>median=31.2 g) | 3658 / 23860 | 1.10 (1.07 to 1.14) | <0.001 | 1530 / 34405 | 1.09 (1.04 to 1.15) | <0.001 |
| In ever smokers | 3607 / 22037 | 1.11 (1.07 to 1.15) | <0.001 | 1677 / 33223 | 1.06 (1.01 to 1.11) | 0.01 |
| In never smokers | 3456 / 25937 | 1.11 (1.07 to 1.15) | <0.001 | 1389 / 35582 | 1.11 (1.06 to 1.17) | <0.001 |

CI: confidence interval, HR: Hazard ratio, SFA: saturated fatty acids, N = 55,307 for overweight analyses and 71,871 for obesity analyses
^*^ HR for an absolute increment of 10 in the percentage of ultra-processed food in the diet out of the total quantity of food consumed, except when stated otherwise

^a^ Models were adjusted for age (timescale), sex (except when stratified), educational level (<high school, <2 years after school, ≥ 2 years after high school), marital status (living alone or not), baseline BMI, physical activity (high, moderate, low), smoking status (never, former, current/except when stratified), alcohol intake, number of 24h-dietary records and energy intake
^b^ Multiple imputation for missing data using the MICE method [11] by fully conditional specification (FCS, 20 imputed datasets) for level of education and physical activity level. Results were combined across imputation based on Rubin’s combination rules [12,13] using the SAS PROC MIANALYZE procedure [14].

^c^ Participants with missing data for level of education and physical activity level were excluded

| **Table B** – Sensitivity analyses: Associations between the quantity of each ultra-processed food group for an increase of 100g/d, and the risks of overweight and obesity, NutriNet-Santé cohort, France, 2009-2019^a^   \|  \| **Overweight risk** \| \| \| **Obesity risk** \| \| \| \| --- \| --- \| --- \| --- \| --- \| --- \| --- \| \|  \| **N cases/non-cases** \| HR* (95% CI) \| p-value \| **N cases/non-cases** \| HR* (95% CI) \| p-value \| \| \| Overall amount of UP foods and drinks \| 7063 / 47974 \| 1.04 (1.03 to 1.03) \| <0.001 \| 3066 / 68805 \| 1.05 (1.03 to 1.06) \| <0.001 \| \| \| UP beverages \| 7063 / 47974 \| 1.04 (1.03 to 1.15) \| <0.001 \| 3066 / 68805 \| 1.06 (1.05 to 1.08) \| <0.001 \| \| \| UP dairy products \| 7063 / 47974 \| 1.09 (1.05 to 1.12) \| <0.001 \| 3066 / 68805 \| 1.08 (1.02 to 1.13) \| 0.004 \| \| \| UP fats and sauces \| 7063 / 47974 \| 1.23 (1.12 to 1.50) \| <0.001 \| 3066 / 68805 \| 1.26 (1.03 to 1.54) \| 0.02 \| \| \| UP fruits and vegetables \| 7063 / 47974 \| 0.98 (0.96 to 1.01) \| 0.1 \| 3066 / 68805 \| 1.00 (0.97 to 1.03) \| 0.9 \| \| \| UP meat, fish and egg \| 7063 / 47974 \| 1.30 (1.22 to 1.38) \| <0.001 \| 3066 / 68805 \| 1.16 (1.06 to 1.27) \| 0.002 \| \| \| UP starchy foods and breakfast cereals \| 7063 / 47974 \| 1.07 (1.01 to 1.13) \| 0.03 \| 3066 / 68805 \| 1.07 (0.98 to 1.17) \| 0.1 \| \| \| UP sugary products \| 7063 / 47974 \| 1.00 (1.00 to 1.01) \| 0.2 \| 3066 / 68805 \| 1.00 (0.99 to 1.00) \| 0.3 \| \| \| UP salty snacks \| 7063 / 47974 \| 1.01 (0.84 to 1.23) \| 0.8 \| 3066 / 68805 \| 1.12 (0.83 to 1.51) \| 0.5 \| \| |  |  |  |  |  |  |
| --- | --- | --- | --- | --- | --- | --- | --- | --- | --- | --- | --- | --- | --- | --- | --- | --- | --- | --- | --- | --- | --- | --- | --- | --- | --- | --- | --- | --- | --- | --- | --- | --- | --- | --- | --- | --- | --- | --- | --- | --- | --- | --- | --- | --- | --- | --- | --- | --- | --- | --- | --- | --- | --- | --- | --- | --- | --- | --- | --- | --- | --- | --- | --- | --- | --- | --- | --- | --- | --- | --- | --- | --- | --- | --- | --- | --- | --- | --- | --- | --- | --- | --- | --- | --- | --- | --- | --- | --- | --- | --- | --- | --- | --- |

CI: confidence interval, HR: Hazard ratio, UP: Ultra-processed, N = 55,307 for overweight analyses and 71,871 for obesity analyses

*HR for an absolute increment of 100 g/day in the consumption of the ultra-processed food group
^a^ Models were adjusted for age (timescale), sex, educational level (<high school, <2 years after school, ≥ 2 years after high school), marital status (living alone or not), baseline BMI, physical activity (high, moderate, low), smoking status (never, former, current), alcohol intake, number of 24h-dietary records and energy intake

**References**

1. Black AE. Critical evaluation of energy intake using the Goldberg cut-off for energy intake:basal metabolic rate. A practical guide to its calculation, use and limitations. Int J Obes Relat Metab Disord. 2000;24: 1119–1130.

2. Black AE. The sensitivity and specificity of the Goldberg cut-off for EI:BMR for identifying diet reports of poor validity. Eur J Clin Nutr. 2000;54: 395–404.

3. Goldberg GR, Black AE, Jebb SA, Cole TJ, Murgatroyd PR, Coward WA, et al. Critical evaluation of energy intake data using fundamental principles of energy physiology: 1. Derivation of cut-off limits to identify under-recording. Eur J Clin Nutr. 1991;45: 569–581.

4. Schofield WN. Predicting basal metabolic rate, new standards and review of previous work. Hum Nutr Clin Nutr. 1985;39 Suppl 1: 5–41.

5. Monteiro CA, Cannon G, Levy RB, Moubarac JC, Jaime PC, Martins AP, et al. NOVA. The star shines bright. World Nutrition. 2016;7: 28–38.

6. Monteiro CA, Cannon G, Moubarac JC, Levy RB, Louzada ML, Jaime PC. The UN Decade of Nutrition, the NOVA food classification and the trouble with ultra-processing. Public Health Nutr. 2017; 1–13. doi:10.1017/S1368980017000234

7. Moubarac JC, Parra DC, Cannon G, Monteiro CA. Food Classification Systems Based on Food Processing: Significance and Implications for Policies and Actions: A Systematic Literature Review and Assessment. Curr Obes Rep. 2014;3: 256–272. doi:10.1007/s13679-014-0092-0

8. Julia C, Martinez L, Alles B, Touvier M, Hercberg S, Mejean C, et al. Contribution of ultra-processed foods in the diet of adults from the French NutriNet-Sante study. Public Health Nutr. 2017; 1–11. doi:10.1017/S1368980017001367

9. Craig CL, Marshall AL, Sjostrom M, Bauman AE, Booth ML, Ainsworth BE, et al. International physical activity questionnaire: 12-country reliability and validity. Med Sci Sports Exerc. 2003;35: 1381–1395. doi:10.1249/01.MSS.0000078924.61453.FB

10. IPAQ Group. Guidelines for Data Processing and Analysis of the International Physical Activity Questionnaire (IPAQ). 2005.

11. van BS. Multiple imputation of discrete and continuous data by fully conditional specification. Stat Methods Med Res. 2007;16: 219–242. doi:10.1177/0962280206074463

12. Rubin DB. Inference and missing data. Biometrika. 1976;63: 581–592. doi:10.1093/biomet/63.3.581

13. Rubin DB. Multiple Imputation for Nonresponse in Surveys. John Wiley & Sons; 2004.

14. PROC MIANALYZE: The MIANALYZE Procedure :: SAS/STAT(R) 9.2 User’s Guide, Second Edition. [cited 19 Dec 2018]. Available: https://support.sas.com/documentation/cdl/en/statug/63033/HTML/default/viewer.htm#mianalyze_toc.htm
