# Supplementary material for: Ultra-processed food intake in association with BMI change and risk of overweight and obesity: A prospective analysis of the French NutriNet-Santé cohort
Source: PLoS Med. 2020 Aug 27;17(8):e1003256. doi: 10.1371/journal.pmed.1003256 (PMC7451582; doi:10.1371/journal.pmed.1003256)
Supplement: Attachment — Submitted filename: R1_SROUR_upfweight_response_final.docx [file pmed.1003256.s003.docx]

We would like to sincerely thank the Editors and the reviews for granting us the opportunity to revise this paper. Their valuable comments helped us improve substantially the scientific quality and the readability of the paper. Kindly find below our point-by-point response.

Requests from the editors:

*In the abstract (below), suggest clarify the meaning of the figures in brackets - assume these are 95%CI but that should be stated. "UPF intake was associated with a higher risk of overweight (n = 7,063 overweight participants; hazard ratio for an absolute increase of 10% of ultra-processed foods in the diet = 1.11 (1.08-1.14); P<0.0001) and obesity (n = 3,066 incident obese participants; HR10% = 1.09 (1.05-1.13); P<0.0001)."

**This has been clarified in the abstract page 2.

*In the last sentence of the Abstract Methods and Findings section, please summarise any key main limitation(s) of the study's methodology (currently there is a good detailed description of this in the main text).

**We have summarized the main limitations at the end of the Results section. It now reads:

*“**Study limitations include possible selection bias, potential residual confounding due to the observational design, and a possible item-misclassification according to the level of processing. Nonetheless, robustness was tested and verified using a large panel of sensitivity analyses.”*

*At this stage, we ask that you include a short, non-technical Author Summary of your research to make findings accessible to a wide audience that includes both scientists and non-scientists. The Author Summary should immediately follow the Abstract in your revised manuscript. This text is subject to editorial change and should be distinct from the scientific abstract. Please see our author guidelines for more information: <https://journals.plos.org/plosmedicine/s/revising-your-manuscript#loc-author-summary>

**A non-technical Author summary has been added.

***Why Was This Study Done?***

- *Ultra-processed food (UPF) consumption has increased drastically worldwide and already represents 50-60% of total daily energy intake in several high-income countries*
- *These changes in dietary behaviours are concomitant with a continuous rise in the prevalence of overweight and obesity during the last century*
- *Only two prospective studies investigated the associations between UPF consumption and overweight/obesity risks*
- *We studied the associations between the contribution of UPF to the diets of more than 100,000 participants and longitudinal changes in BMI, and risks of overweight and obesity*

***What Did the Researchers Do and Find?***

- *We used appropriate statistical models to study the associations between UPF consumption and BMI change, and risks of overweight and obesity in the NutriNet-Santé cohort*
- *110,260 participants were followed between 2009 and 2019*
- *Having a higher consumption of UPF was associated with an increased weight gain, as well as increased risks of becoming overweight or obese*
- *These associations were not fully explained by the overall poorer nutritional quality of UPF*

***What Do These Findings Mean?***

- *These results suggest that consumption of UPF is associated with increased weight gain*
- *This study contributes to the mounting evidence on the link between food processing and health*
- *Further studies (epidemiological and experimental) are needed to investigate the relative contribution of nutritional composition, food additives, process- or packaging-related contaminants and modification of the food matrix*
- *UPF consumption should be limited and the consumption of unprocessed or minimally processed foods should be promoted instead, as several national nutritional policies recommend*

*If possible, please reformat in-text reference callouts to use numbers in square brackets (this should be quick and easy if referencing software was used). Many thanks

**The reference style has been modified to match the required format.

*Main text, main sections should be Introduction, Methods (not Materials and Methods), Results, Discussion.

** “Materials and Methods” has now been replaced by “Methods”.

*Did your study have a prospective protocol or analysis plan? Please state this (either way) early in the Methods section.

a) If a prospective analysis plan (from your funding proposal, IRB or other ethics committee submission, study protocol, or other planning document written before analyzing the data) was used in designing the study, please include the relevant prospectively written document with your revised manuscript as a Supporting Information file to be published alongside your study, and cite it in the Methods section. A legend for this file should be included at the end of your manuscript.

b) If no such document exists, please make sure that the Methods section transparently describes when analyses were planned, and when/why any data-driven changes to analyses took place.

c) In either case, changes in the analysis-- including those made in response to peer review comments-- should be identified as such in the Methods section of the paper, with rationale.

**The end of the first paragraph of the Methods section line 163 (page 5) now reads:

*“**The NutriNet-Santé study is conducted according to the Declaration of Helsinki guidelines and was approved by the Institutional Review Board of the French Institute for Health and Medical Research (IRB Inserm n°0000388FWA00005831) and the "Commission Nationale de l’Informatique et des Libertés" (CNIL n°908450/n°909216). The study is registered at clinicaltrials.gov as NCT03335644. Some other information can be accessed on the website* [*https://info.etude-nutrinet-sante.fr/en*](https://info.etude-nutrinet-sante.fr/en)*. Electronic informed consent was obtained from each participant. All methods have been described in line with the Strengthening the Reporting of Observational Studies in Epidemiology (STROBE) Statement (see STROBE Checklist).”*

The statistical analysis section page 6 lines 223-228 now reads:

*“**Analyses for this specific article were hypothesis-oriented in order to investigate the relationship between UPF consumption and weight gain or prospective occurrence of overweight or obesity. No specific analysis plan has been pre-published for the present article but all analyses were pre-planned by the authors at the time of conception and design of the present study, except one non-prespecified analysis that was performed to comply with peer-review requirements (Mixed models with continuous exposure).”*

*As the paper reports findings from an observational cohort (prospective), we'd suggest using the STROBE guideline to enhance reporting - please include the completed STROBE checklist as Supporting Information. Please add the following statement, or similar, to the Methods: "This study is reported as per the Strengthening the Reporting of Observational Studies in Epidemiology (STROBE) guideline (SChecklist)." The STROBE guideline can be found here: <http://www.equator-network.org/reporting-guidelines/strobe/>. When completing the checklist, please use section and paragraph numbers, rather than page numbers.

**All methods have been described in line with the Strengthening the Reporting of Observational Studies in Epidemiology (STROBE) Statement (see STROBE Checklist). This statement has been added in the Methods line 170.

-----------------------------------------------------------

Comments from the reviewers:

Reviewer #1: Dear editor,

initially I would like to thank you for the invitation to review the manuscript.

This is a cohort study that aimed to investigate the association between the consumption of ultra-processed foods and the risk of overweight and obesity. The article is very well written, clearly presents its relevance and the methods were presented in a detailed way. The authors have considered the possible confounding variables in the adjustment of the models. The results were also clearly exposed. Thus, the publication of the article is recommended because it is a longitudinal population-based study, and there are few studies with this design so far that have analyzed this association between the consumption of ultra-processed foods and the risk of obesity. Only a few points were highlighted that should be further clarified in the text.

**We would like to sincerely thank Reviewer 1 for these positive comments and inputs.

Introduction:

-	line 68: replace today for "nowadays"

** “Today” has now been replaced by “nowadays”.

-	Methods:

Did the study population come from all regions of France?

**Participants come from all regions of France. This has been clarified in the Methods section line 153.

It is not clear if the consumption data were collected only at baseline (three records) or if these data were obtained every six months as the anthropometric data. Please explain better in the text.

** Only baseline dietary data were used to comply with the prospective design and ensure that exposure precedes the outcome. This has been clarified in the Methods section page 5 line 175. It now reads:

*“Dietary data were collected at baseline using a kit of three non-consecutive web-based 24-hour records, randomly assigned over a two-week period (two during weekdays and one during the weekend).”*

Make clear the age range of study participants. Were adults only?

***Participants were adults only, aged between 18.0 and 73.3 years old.* This has been added to the Methods section line 230.

Results:

-	table 1: quartile 4 line 6: replace 16680 for 16,680

**The number 16680 was replaced by 16,680.

-	line 222: "….and with a higher physical activity level." But in table 1 shows that prevalence of high level of PA is higher in the first quartile.

**We apologize for this mistake. This has been amended in the Results section that now reads *"….and with a lower physical activity level."*

-----------------------------------------------------------

Reviewer #2: I restrict my remarks to statistical aspects of this paper. Although much of what was done is good, I have some issues to resolve before I recommend publication.

**We thank Reviewer 2 for his insights.

-	Lines 146-152	BMI is a poor measure of obesity. See e.g. my blog post <https://medium.com/peter-flom-the-blog/why-bmi-is-a-bad-measure-of-obesity-and-what-is-better-f8a62fc9ca49> Change in BMI at least controls for most of the peoblems. But change in BMi obscures whether the person is obese at all. A small increase in BMI for someone who is normal weight or even underweight may not be problematic. This sort of thing is hard to solve, given the failure to get good day at the start (because good data is hard) but it should be mentioned.

**We agree that BMI presents limitations as a proxy for adiposity. Unfortunately, other indicators such as the Relative Fat Mass [1] or waist circumference were not available prospectively in the cohort and thus could not be used in the present analysis. Thus, this analysis was based on BMI, which is still the indicator used in official WHO’s definition of overweight and obesity. We have added this limitation in the discussion section page 15 (line 382) and referenced the blog post suggested by Reviewer 2.

-	Line 177-178 Don't categorize independent variables In ***Regression Modelling Strategies*** Frank Harrell lists 11 problems with this and summarizes "Nothing could be more disastrous". I wrote another blog post <https://medium.com/@peterflom/what-happens-when-we-categorize-an-independent-variable-in-regression-77d4c5862b6c> showing, graphically, the things that can happen. Leave UPF continuous and use splines to investigate nonlinearitiy

Same for line 183 and 193

**The didactic blog recommended by Reviewer 2 was a very inspiring source of information, thank you for bringing it to our knowledge.

In order to comply with Reviewer 2’s comment, we have now added a continuous model for BMI change (in the Methods, Table 2 and the Results section). Furthermore, we checked the linearity assumptions of our Cox models where the continuous exposure was used, using Martingale residuals (Appendix 5), and no violation was detected. These changes have been highlighted in the abstract, and throughout the manuscript and the supplemental material.

As previously done in a paper recently published by our team in Plos Medicine [2], we also kept the results in quartiles (which were consistent with those based on continuous models) for comparison with previous studies on UPF and weight variation that were based on quartiles [3,4], for direct integration in future highest versus lowest meta-analyses [5–8], and for clarity of the presentation of the results for the scientific community, the public health stakeholders and the citizens (e.g. Figure 2 clearly showing the different trajectories by quartiles of UPF).

-	Table 1 - delete the p value column. P values are not relevant here, effect size is. With these N, even small differences are sig. (NOTE: It is OK to make a table liuke this with quartiles, just don't use quartiles in analysis. However density plots would be better here.

**P-values were dropped from Table 1 and a density plot of the distribution of UPF proportion in the diet in the study sample has been added to appendix 2.

Peter Flom

-----------------------------------------------------------

Reviewer #3: Brazil, May 6th 2020

Manuscript Title: Ultra-processed food intake and weight change, overweight and obesity - Findings from the prospective NutriNet-Santé cohort

Manuscript number: PMEDICINE-D-20-00661R1

Comments to the Author

Overall, this paper is important to the community of researchers in this general area and has technical quality. The results provide a substantial advance over existing knowledge, with clear implications for public health. However, the way the paper is currently written, with much of the methodological detail and appendix missing, it is difficult to assess the methodological approach.

**We thank Reviewer 3 for this overall positive comment. Please find below our responses with additional information and clarifications.

Abstract

-	Line 45: the number >3500 refers to the number of items of the 24h dietary records or the number of foods in a food composition table as presented at material and methods section in line 130?

**We have now rephrased Line 45 to clarify this statement. It now reads:

*“Dietary intakes were collected using repeated and validated 24h dietary records linked to a food composition database that included >3,500 different food items, each categorized according to their degree of processing by the NOVA classification.”*

-	Line 58-61: How about weight change? include more information about weight change on the conclusion - may be useful.

**We have included this information in the abstract’s conclusion as requested. It now reads:

*“In this large observational prospective study, higher consumption of UPF was associated with gain in BMI and higher risks of overweight and obesity.”*

Introduction

- Overall, the introduction needs to be revised to incorporate other aspects of the complexity of overweight, obesity and weight change. The authors can then incorporate the literature on food access, for example. There are complex interactions between biological, behavioral, social and environmental factors that are involved in overweight, obesity and weight change; they need to be contemplated.

**We have now incorporated a paragraph on the interactions between behaviors, environmental factors and food access, in the obesity epidemic, line 115-121. It reads:

*“**In addition, drivers of the global obesity epidemic might reside in the change in social behaviours and environmental factors (such as built environment) [9]. Changes in the food system are likely to play a key role in the obesity pandemic [10]: they are notably characterized by increased supply of affordable, hyperpalatable energy-dense food products; along with sophisticated distribution systems to improve accessibility and convenience; and intensive food marketing campaigns. These trends in the food systems were accompanied by major dietary changes in the last decades.”*

- The author highlights the issue of obesity as an important risk factor for metabolic diseases. I suggest that the author reflects: "Is consumption of AUP a risk factor for another risk factor? Or a risk factor for a metabolic disease (ICD-10 code E66) that is simultaneously related to other chronic non-communicable diseases? How can the study of obesity as a risk factor minimize the results found here?

**While obesity is a health outcome itself (studied as such in the present analysis), it is also a major risk factor of metabolic diseases such as coronary heart disease, ischemic stroke and type 2 diabetes [11] but also many cancers [12,13]. Studying the associations between UPF consumption and obesity is relevant as it is, given the metabolic and economic burdens of obesity; but it is also relevant given the role of obesity as a risk factor for these other chronic diseases. In one of our previous prospective studies, we found that UPF consumption was associated with an increased risk of type-2 diabetes [14], and that this association was not fully explained by weight gain. We also found associations between UPF consumption and overall cancer risk, and CVD risk in non-obese participants [15,16]. UPF could therefore play both, a direct role in the development of CVD, cancer, and T2D, as discussed elsewhere, but also an indirect role through weight gain and obesity as we observe in the present study. This double pathway is already know and well-described for other dietary factors, such as fruit and vegetables, a protector against head-and-neck cancers with a probable level of evidence according to the World Cancer Research Fund [13] through both a direct effect (i.e. DNA methylation, redox status) and an indirect effect through decreasing overweight and obesity risks [13]. These points have been added to the discussion pages 14-15 (lines 354-368).

- In addition to the lack of prospective studies, explicit the study's contribution to the state of the art on the subject.

**The study’s contribution has been clarified. The end of the introduction now reads:

*“Consistently, several cross sectional and ecological studies have substantiated a positive association between UPF consumption and obesity [19,31–35], but prospective studies are lacking as only two of them, one in Spain [36] and one in Brazil [37] were conducted; both relying on dietary data from Food Frequency Questionnaires (FFQ), studying the contribution of UPF to the energy intake, and therefore not accounting for low-calorie and artificially sweetened products.*

*This largescale prospective study aimed to investigate the associations between UPF consumption and weight change, as well as the risk of overweight and obesity, among adults from the large-scale NutriNet-Santé cohort.”*

Material and methods

-	In the methodology, additional details regarding process of recruitment are needed. Is there any possible implications of this selection for the evidence produced by the study? I realize that additional details have already been published but it is hard to understand the process without a few more specific details.

**Details regarding enrolment in the cohort have now been added, page 5.

*Briefly, participants from all regions of France with access to the internet have been continuously recruited, on a voluntary basis, from the general population since May 2009. Vast multimedia campaigns (television, radio, national and regional newspapers, posters, internet) called for volunteers by providing details on the study's specific web-site where volunteers can subscribe. A relay of information was also maintained on a large number of websites (national institutions, city councils, private firms, web organisations). A billboard advertising campaign was also available through professional channels (doctors, pharmacists, dentists, business partners, municipalities, etc.)* [17]*.*

As other volunteer-based cohorts, and as stated in the Limitations section lines 369-372 (page 15), self-selection may have led to the inclusion of subjects with a higher socio-economic position [18] and who were more concerned with their diets, and with healthier dietary patterns than in the general population [19]. We might therefore have underestimated the studied associations due to a lower contrast between extreme quartiles of UPF consumption.

-	In the data collection, can the authors be more specific about the number of waves that the cohort presents, the time difference between them and whether the variable outcome and explanatory were measured in all waves?

**Three non-consecutive dietary records administered at baseline were used to determine the exposure of interest (% of UPF in the diet) in this study. Anthropometric data was collected at baseline and every 6 months thereafter between May 2009 and June 2019. More generally, the online NutriNet-Santé platform is designed to send an average of one questionnaire per month, allowing us to collect additional information on various research topics (e.g. sleep duration, environmental exposures, mental health, cooking practices). This has been clarified in the Methods section, lines 175-176, 201-202 and 162-163.

-	Line 128-129: Can the authors expand why dietary intakes from the baseline 24-hour dietary records were considered as usual dietary intakes? What are the limitations to this premise?

**Multiple non-consecutive dietary records for the same individual are considered a valid tool to assess the usual diet [20,21]. We chose to focus on baseline records in this analysis in order to comply with the prospective design and ensure that exposure (UPF consumption) precedes the outcome (BMI change of incident obesity/overweight). This design limits the risk of reverse causality, where participants would modify their dietary intakes due to their weight change. It cannot be excluded however that weight changes occurring late during follow-up may be related to later dietary changes rather than to baseline diet; this probably tended to decrease the strength of the associations observed in this study. This has been clarified in the discussion page 15 (lines 387-393).

-	Line 131: Can the authors be more specific about the method proposed by Black? What characterizes "under-reporters"? What were these definitions based on? I realize that additional details have already been published but it's hard to understand the exclusion without a few more specific details. And how about over-reporters? They were excluded?

**Dietary underreporting was identified with the method proposed by Black, using the basal metabolic rate and Goldberg cut-off, in order to screen participants with abnormally low energy intakes, and energy under-reporters (20.0% of the cohort) were excluded [22]. This has been stated in the Methods section page 5 (lines 183-186) and detailed methodology for under-reporting is now presented in Appendix 1. No exclusion was made for high energy intake (Highest energy intakes corresponded to plausible values (99th percentile = 3600 kcal/d) and thus were kept for analysis).

-	Line 146: And in the "Obesity and overweight" subsection how was the incidence of overweight and obesity calculated.

**The absolute incidence rates of Obesity and overweight were not calculated as this was not the objective of the analyses. In the proportional risk models used, the hazard ratios represented the relative rate of instantaneous risk of overweight or obesity between different groups of consumption of UPF. In order to avoid this confusion, we have now replaced “incidence of obesity or overweight” by “risk”, in the abstract and throughout the manuscript.

-	I suggest a subsection entitled "Weight change" (before or after obesity and overweight) or complete the subsection "Obesity and overweight". How was the weight change assessed? Delta? % weight loss? MBI change as present on Line 182? This is one of the outcomes of this study and its calculation must be detailed. Finally, if this outcome was assessed as a change in BMI, it is important to clarify it in the title and throughout the manuscript.

**We have now replaced weight change by BMI change in the title, abstract and throughout the manuscript where appropriate. As for Line 182, this section is dedicated to data collection and not data analysis; we have now renamed it to “Anthropometric data”.

As stated in Line 256, the modelled outcome was the absolute change (∆) in BMI, assessed thanks to a mixed model for repeated data, with a random effect on intercept and time and a repeated effect on individuals. This model also allows for adjustment for several confounders as it is a regression model.

-	159-160: Can the authors be more specific about the categories of low, moderate and high physical activity levels? It is suggested to insert the cutoff points.

**Details of the computation of the physical activity levels are now displayed in Appendix 3.

-	Include indicators of the nutritional quality of the diet (alcohol, sodium, saturated fatty, dietary fiber and sugar intake, number of dietary records, fruit and vegetables, sugary drinks, Healthy and Western dietary patterns) as covariates. They were presented only in the tables and not detailed in the methods. Please, how were they obtained?

**These indicators have now been included in the Covariates section, which now reads:

*“Several indicators of the nutritional quality of the diet were also computed based on average dietary intakes from baseline 24h dietary records and were used as covariates: daily nutrient intakes (sugar, fibre, sodium, and saturated fatty acid (SFA)) calculated using the food composition database; consumptions of several food groups (fruit, vegetables, and sugary drinks); and Healthy and Western dietary patterns, derived from Principal Component Analysis (see Appendix 4).”*

-	I suggest the exclusion of covariates present at that section, but not applied in the statistical analyses or adjustment, for example: time spent for screen watching and sedentary behaviors.

**Time spent for screen watching and sedentary behaviors was indeed used as an adjustment variable in sensitivity analyses (appendix 6).

-	Line 165: Can the authors explain why exclude participants with missing anthropometric in place of apply a method of processing these missing data, for example imputation - as done for covariates (Line 178-182)? Do individuals excluded because of this information differ from those included in the study?

**The outcome of the present study (BMI) is self-reported. We have therefore made the assumption that missing data for weight report would not be completely at random, and that participants having gained more weight would be more incline to not answer the follow-up anthropometric questionnaires, for social desirability purposes [23]. Applying multiple imputation (as performed for covariates in this study) would therefore not be suitable, and participants (non-cases) were right-censored at the date of their last filled anthropometric questionnaire.

-	It isn't clear how sex-specific quartiles were accessed. That detail can lead the reader to better understand how only one data for sex-quartile were presented per quartile on table 1 for example.

**Quartile cut-offs were calculated separately in men and women based on the respective distribution of UPF consumption. Men on the one hand and women on the other hand were therefore categorized into one of the quartiles based on their UPF consumption. Next, quartiles 1 in men and women were merged; identically for quartiles 2, 3 and 4. This classical procedure was automatically performed using SAS proc rank by sex. This has been clarified in the Methods section page 6 (lines 243-245).

-	Can the authors clarify why adjust for sex if the proportion of UPF in the diet was sex-specific quartiles?

**We adjusted for sex because we assumed that other covariates might also depend on sex, and not only UPF consumption. Leaving the models unadjusted for sex could therefore introduce residual confounding.

-	Can the authors clarify why adjust for Healthy and Western dietary patterns and consumption of fruit and vegetables? Fruit and vegetables consumption do not also refers to a Healthy dietary patterns?

**We have chosen this adjustment strategy to have both a model adjusted for the overall quality of the diet, using dietary patterns (Model 3), and a model (Model 4) adjusted for individual food groups (fruit, vegetables and sugary drinks) for which a strong level of evidence as risk or protective factors of weight gain has been established by the World Cancer Research Fund. As stated in the footnotes, these models were not nested: models adjusted for food groups were unadjusted for patterns.

-	Improve the writing of the additional adjustments for sugar, fiber, sodium, and saturated fatty acid (SFA). Can the authors expand why that adjust were performed?

**We have now clarified the reasons behind these adjustments, page 7 (lines 258-262):

*“Additional adjustments were tested in different models: for sugar, fibre, sodium, and SFA intakes (to adjust for the nutritional quality of UPF), for dietary patterns (to capture the overall quality of the diet), and for consumptions of fruit, vegetables, and sugary drinks (convincingly linked to the risk of weight gain according to the WCRF/AICR)* [13]*”.*

-	Line 202-2012: Improve the wording of the two last paragraphs of the statistical analyses. It is confusing. Perhaps, highlight that it refers to Secondary and sensitivity analyses.

**We have now added a subtitle for this section “Sensitivity and Secondary analyses” and clarified the wording line 274.

-	Line 202-204: Can the authors clarify why reverse causality is a problem in this study. It is not a prospective?

**Reverse causality might be a problem if participants exhibiting weight change try to modify their dietary habits. As stated above, guaranteeing that dietary exposure (at baseline) precedes the outcome (prospective design) allows limiting this bias. This has been clarified in the manuscript page 15 (lines 387-393).

Results

-	Much of the information in the tables were not included or explained in the Material and Methods section. This should be reorganized. For example, additional BMI gain, Time…

**This has been checked and amended. Notably Time has now been clarified in the Methods section page 7(line 254), and additional BMI gain in the footnotes to Table 2.

-	Figure 1: separate the exclusions of those who were "overweight or obese" from those "missing from anthropometry".

**The exclusions have been separated and figure 1 has been modified accordingly.

-	Table 1: The Sex-specific quartiles of the proportion of ultra-processed food intake in the total quantity of food consumed were accessed for all quartiles, correct? So include letter b for all quartiles or clarify the note for example: Cut-offs for quartiles were 10.2, 15.5 and 22.5 for men and 9.9, 15.2 and 22.1 for women, respectively.

**This modification has been made.

-	Table 1 Note: Please add the full IPAQ name and Standard Deviation (SDs). And a brief explanation about IPAQ categories (low, moderate and High). The table must be self-explanatory.

**This modification has been made.

-	Table 2 - Standardize the number of decimal places to the p value

**Plos Medicine has no specific instructions for number of decimal places to the p-value. We therefore made the choice to stop at the first non-null decimal for p>0.0001; and report p<0.0001 for smaller values.

-	Table 2 Note: Please include the definition of the abbreviation for BMI.

**This modification has been made.

-	Table 3 - Standardize the number of decimal places to the p value

**As stated above, Plos Medicine has no specific instructions for number of decimal places to the p-value. We therefore made the choice to stop at the first non-null decimal for p>0.0001; and report p<0.0001 for smaller values.

-	Table 3 Note: Please include the definition of the abbreviation for Q1, Q2, Q3 e Q4.

**This modification has been made.

Discussion

-	Exclude line 319, 354 and 374

**These lines have been excluded.

-	296-300: I suggest rephrasing or deleting these phrases to better fit the population (adults), object of study of the manuscript.

**These phrases were now deleted.

-	The presentation of non-nutritional bioactive compounds as a hypothesis for the associations found is fantastic and real. But how these findings might inform policies and/or programming? It is urgent to include aspects of environment on that discussion.

**A paragraph about these aspects, and the potential of the study has been included page 15 (lines 398-405). It reads:

*“Political discussions are currently ongoing in France and in Europe to decrease the number of authorized food additives. Therefore, this study contributes to the mounting level of evidence on food processing and human health, needed by public policies to update dietary guidelines in the future, by integrating aspects of food processing, and potentially regulating more-tightly the policies related to food additives once high-quality results become available. In Brazil for instance, health authorities recently decided that at least 75% of the budget of the Brazilian School Meal Program must be used to buy unprocessed or minimally processed foods, and maximum 20% to buy processed or ultra-processed foods* [24]*.”*

The discussion would benefit from integrating the findings with additional existing literature about obesogenic environment, which would connect UPF consumption and obesity. Specially on the second paragraph in which the author highlights the nutritional quality of UPF does not appear to be entirely responsible association with weight gain.

**We have now integrated aspects about obesogenic environment in the Discussion page 14 (lines 348-353). The sentence reads:

*“Besides, the increased availability, accessibility and affordability of these UPF on the market, in addition to their excessive marketing, might at least partly play a role, even though we adjusted for energy intake, in these associations, as it contributes to an obesogenic environment* [9,25,26]*. Therefore, it is important to act on slowing down the obesity epidemic not only through dietary guidelines, but also by ensuring the availability of healthy products and the establishment of an environment that encourages healthy behaviours.”*

-	Line 355: replace "First" for "first".

**We have added a comma before “First”.

-	The sentences "[…] However, this would have led to a non-differential measurement error (in cases and non-cases), probably biasing results towards the null hypothesis. Moreover, despite adjustment for an extended range of cofactors and stratified analyses, residual confounding cannot be entirely ruled out, thus no causality can be established […]". They are very emphatic. Causality must be established with caution, but not established.

**We have now replaced “no causality can be established” by “caution is needed when making causal inference.”

-	The author should exclude the self-reported anthropometric as a limitation, once the data has been validated (Lassale C, Péneau S, Touvier M, Julia C, Galan P, Hercberg S, et al. Validity of web-based self551 reported weight and height: results of the Nutrinet-Santé study. J Med Internet Res. 2013 Aug 552 8;15(8):e152)

**This limitation has been excluded.

-	The author should include a paragraph with the potential of the study.

**As stated above, a paragraph about these aspects, and the potential of the study has been included in page 15. It reads:

*“Political discussions are currently ongoing in France and in Europe to decrease the number of authorized food additives. Therefore, this study contributes to the mounting level of evidence on food processing and human health, needed by public policies to update dietary guidelines in the future, by integrating aspects of food processing, and potentially regulating more-tightly the policies related to food additives once high-quality results become available. In Brazil for instance, health authorities recently decided that at least 75% of the budget of the Brazilian School Meal Program must be used to buy unprocessed or minimally processed foods, and maximum 20% to buy processed or ultra-processed foods* [24]*.”*

-	The conclusions would benefit from providing some insight of how these findings might inform policies and/or programming and with broader implications of the study findings.

**We have provided some insights about this in the end of our conclusion. It now reads:

*“The accumulation of consistent findings on the link between UPF and health, which this study contributes to, along with the environmental non-sustainability of these products* [27] *is leading national public health authorities, in France and Brazil for instance* [28–30]*, to recommend privileging the consumption of unprocessed/minimally processed foods, and limiting the consumption of UPF. The French National Programme for Nutrition and Health (PNNS) has set a target of reducing the consumption of UPF in France of 20% by 2022, and the Brazilian School Meal Program is going towards limiting UPF purchases* [24]*. In addition, these findings might help physicians and dietitians in clinical practice by providing evidence about the role of UPF in weight gain and obesity management.”*

Reference list

- Add space between the words that make up the name of scientific journals

#8 Obes Rev.

#9: Int J Behav Nutr Phys Act.

#15 and #19: Rev Saude Publica

#17 and #35: Am J Clin Nutr.

#18: Eur J Clin Nutr

#39: Int J Obes Relat Metab

#40 and #42: Br J Nutr.

#41: J Acad Nutr Diet.

#48: Int J Public Health

#49: Med Sci Sports Exerc.

-#42 and #43 are the same. Exclude one of them and review citations numbers in the manuscript.

**Spaces have been added, and duplicates have been deleted.

-----------------------------------------------------------

Any attachments provided with reviews can be seen via the following link:

References

1.	Woolcott OO, Bergman RN. Defining cutoffs to diagnose obesity using the relative fat mass (RFM): Association with mortality in NHANES 1999-2014. Int J Obes (Lond). 2020. doi:10.1038/s41366-019-0516-8

2.	Chaltiel D, Julia C, Adjibade M, Touvier M, Hercberg S, Kesse-Guyot E. Adherence to the 2017 French dietary guidelines and adult weight gain: A cohort study. PLOS Medicine. 2019;16: e1003007. doi:10.1371/journal.pmed.1003007

3.	Canhada SL, Luft VC, Giatti L, Duncan BB, Chor D, Fonseca M de JM da, et al. Ultra-processed foods, incident overweight and obesity, and longitudinal changes in weight and waist circumference: the Brazilian Longitudinal Study of Adult Health (ELSA-Brasil). Public Health Nutrition. undefined/ed; 1–11. doi:10.1017/S1368980019002854

4.	Mendonca RD, Pimenta AM, Gea A, Fuente-Arrillaga C, Martinez-Gonzalez MA, Lopes AC, et al. Ultraprocessed food consumption and risk of overweight and obesity: the University of Navarra Follow-Up (SUN) cohort study. Am J Clin Nutr. 2016;104: 1433–1440. doi:10.3945/ajcn.116.135004

5.	Morenga LT, Mallard S, Mann J. Dietary sugars and body weight: systematic review and meta-analyses of randomised controlled trials and cohort studies. BMJ. 2013;346. doi:10.1136/bmj.e7492

6.	Schwingshackl L, Bogensberger B, Hoffmann G. Diet Quality as Assessed by the Healthy Eating Index, Alternate Healthy Eating Index, Dietary Approaches to Stop Hypertension Score, and Health Outcomes: An Updated Systematic Review and Meta-Analysis of Cohort Studies. Journal of the Academy of Nutrition and Dietetics. 2018;118: 74-100.e11. doi:10.1016/j.jand.2017.08.024

7.	Schwingshackl L, Hoffmann G, Lampousi A-M, Knüppel S, Iqbal K, Schwedhelm C, et al. Food groups and risk of type 2 diabetes mellitus: a systematic review and meta-analysis of prospective studies. Eur J Epidemiol. 2017;32: 363–375. doi:10.1007/s10654-017-0246-y

8.	Collin LJ, Judd S, Safford M, Vaccarino V, Welsh JA. Association of Sugary Beverage Consumption With Mortality Risk in US Adults: A Secondary Analysis of Data From the REGARDS Study. JAMA Netw Open. 2019;2: e193121–e193121. doi:10.1001/jamanetworkopen.2019.3121

9.	Swinburn BA, Sacks G, Hall KD, McPherson K, Finegood DT, Moodie ML, et al. The global obesity pandemic: shaped by global drivers and local environments. The Lancet. 2011;378: 804–814. doi:10.1016/S0140-6736(11)60813-1

10.	Cutler DM, Glaeser EL, Shapiro JM. Why Have Americans Become More Obese? Journal of Economic Perspectives. 2003;17: 93–118. doi:10.1257/089533003769204371

11.	World Health Organization. Factsheet on Obesity and overweight. In: WHO [Internet]. 16 Feb 2018 [cited 3 Dec 2019]. Available: https://www.who.int/news-room/fact-sheets/detail/obesity-and-overweight

12.	Latino-Martel P, Cottet V, Druesne-Pecollo N, Pierre FHF, Touillaud M, Touvier M, et al. Alcoholic beverages, obesity, physical activity and other nutritional factors, and cancer risk: A review of the evidence. Critical Reviews in Oncology/Hematology. 2016;99: 308–323. doi:10.1016/j.critrevonc.2016.01.002

13.	WCRF/AICR. Diet, Nutrition, Physical Activity and Cancer: a Global Perspective. Continuous Update Project Expert Report 2018. Recommendations and public health and policy implications. 2018.

14.	Srour B, Fezeu LK, Kesse-Guyot E, Allès B, Debras C, Druesne-Pecollo N, et al. Ultraprocessed Food Consumption and Risk of Type 2 Diabetes Among Participants of the NutriNet-Santé Prospective Cohort. JAMA Intern Med. 2020;180: 283. doi:10.1001/jamainternmed.2019.5942

15.	Srour B, Fezeu LK, Kesse-Guyot E, Allès B, Méjean C, Andrianasolo RM, et al. Ultra-processed food intake and risk of cardiovascular disease: prospective cohort study (NutriNet-Santé). BMJ. 2019;365: l1451. doi:10.1136/bmj.l1451

16.	Fiolet T, Srour B, Sellem L, Kesse-Guyot E, Allès B, Méjean C, et al. Consumption of ultra-processed foods and cancer risk: results from NutriNet-Santé prospective cohort. BMJ. 2018;360: k322.

17.	Kesse-Guyot E, Andreeva V, Castetbon K, Vernay M, Touvier M, Méjean C, et al. Participant profiles according to recruitment source in a large Web-based prospective study: experience from the Nutrinet-Santé study. J Med Internet Res. 2013;15: e205. doi:10.2196/jmir.2488

18.	Andreeva VA, Salanave B, Castetbon K, Deschamps V, Vernay M, Kesse-Guyot E, et al. Comparison of the sociodemographic characteristics of the large NutriNet-Sante e-cohort with French Census data: the issue of volunteer bias revisited. J EpidemiolCommunity Health. 2015;69: 893–898. doi:10.1136/jech-2014-205263

19.	Andreeva VA, Deschamps V, Salanave B, Castetbon K, Verdot C, Kesse-Guyot E, et al. Comparison of Dietary Intakes Between a Large Online Cohort Study (Etude NutriNet-Santé) and a Nationally Representative Cross-Sectional Study (Etude Nationale Nutrition Santé) in France: Addressing the Issue of Generalizability in E-Epidemiology. Am J Epidemiol. 2016;184: 660–669. doi:10.1093/aje/kww016

20.	Gibney MJ, Nutrition Society (Great Britain). Public health nutrition. Oxford, UK; Ames, Iowa: Blackwell Science; 2004. Available: http://www.123library.org/book_details/?id=64307

21.	Willett W. Nutritional Epidemiology. Oxford University Press; 2012. doi:10.1093/acprof:oso/9780199754038.001.0001

22.	Black AE. Critical evaluation of energy intake using the Goldberg cut-off for energy intake:basal metabolic rate. A practical guide to its calculation, use and limitations. Int J Obes Relat Metab Disord. 2000;24: 1119–1130.

23.	Grimm P. Social Desirability Bias. Wiley International Encyclopedia of Marketing. American Cancer Society; 2010. doi:10.1002/9781444316568.wiem02057

24.	Nacional Imprensa. RESOLUÇÃO N^o^ 6, DE 8 DE MAIO DE 2020 - RESOLUÇÃO N^o^ 6, DE 8 DE MAIO DE 2020 - DOU - Imprensa Nacional. [cited 29 May 2020]. Available: http://www.in.gov.br/web/dou

25.	Hobbs M, Radley D. Obesogenic environments and obesity: a comment on ‘Are environmental area characteristics at birth associated with overweight and obesity in school-aged children? Findings from the SLOPE (Studying Lifecourse Obesity PrEdictors) population-based cohort in the south of England.’ BMC Medicine. 2020;18: 59. doi:10.1186/s12916-020-01538-5

26.	Tyrrell J, Wood AR, Ames RM, Yaghootkar H, Beaumont RN, Jones SE, et al. Gene–obesogenic environment interactions in the UK Biobank study. Int J Epidemiol. 2017;46: 559–575. doi:10.1093/ije/dyw337

27.	Swinburn BA, Kraak VI, Allender S, Atkins VJ, Baker PI, Bogard JR, et al. The Global Syndemic of Obesity, Undernutrition, and Climate Change: The Lancet Commission report. The Lancet. 2019;393: 791–846. doi:10.1016/S0140-6736(18)32822-8

28.	Monteiro CA, Cannon G, Lawrence M, Costa Louzada ML da, Pereira Machado P. Ultra-processed foods,diet quality, and health using the NOVA classification system. Rome, FAO. 2019 [cited 4 Sep 2019]. Available: http://www.fao.org/3/ca5644en/ca5644en.pdf

29.	Haut Conseil de la Santé Publique. Avis relatif à la révision des repères alimentaires pour les adultes du futur Programme National Nutrition Santé 2017-2021. 2017 Feb. Available: http://www.hcsp.fr/Explore.cgi/Telecharger?NomFichier=hcspa20170216_reperesalimentairesactua2017.pdf

30.	Ministry of Health of Brazil. Dietary Guidelines for the Brazilian population. 2014. Available: http://189.28.128.100/dab/docs/portaldab/publicacoes/guia_alimentar_populacao_ingles.pdf
