# Supplementary material for: Ultra-processed food intake in association with BMI change and risk of overweight and obesity: A prospective analysis of the French NutriNet-Santé cohort
Source: PLoS Med. 2020 Aug 27;17(8):e1003256. doi: 10.1371/journal.pmed.1003256 (PMC7451582; doi:10.1371/journal.pmed.1003256)
Supplement: Attachment — Submitted filename: R2_SROUR_Pointbypoint.docx [file pmed.1003256.s004.docx]

We would like to sincerely thank the Editors and the Reviewers for accepting to furtherly revise this manuscript, and for their precious comments that improved the quality of the manuscript. All the comments and queries from the Editors, Reviewers, and the Production team have been assessed. Please find below our point-by-point response.

Requests from Editors:
Can I clarify the research design, please? I suspect that the data have been collected prospectively, but that this analysis is retrospective - you use the word "prospective" twice in the abstract. Please can be clear about the design.
Title- please change from
Ultra-processed food intake in association with BMI change and risk of overweight and obesity - Findings from the prospective NutriNet-Santé cohort
To
Ultra-processed food intake in association with BMI change and risk of overweight and obesity; a prospective analysis of the French NutriNet-Santé cohort
- Indeed, this is a prospective study, since the analysis was based on a prospective cohort, and participants were not selected according to their outcome followed by a retrospective data collection; but rather according to their exposure at the time of enrollment in the cohort and they were followed, and the occurring outcomes (overweight, obesity, change in BMI) were assessed using survival analyses and mixed models. We have now clarified this in the abstract; and the title was changed as requested.
Please add aggregate demographic details to go in the abstract; adjustment factors should be listed
- Demographic details and adjustment factors have now been added as requested.
- p<0.0001 -> p<0.001 please
- This was changed throughout the abstract, manuscript, and Appendix.
THE STROBE – can I just query the symbols used. Noting that sections and paragraphs should be used.
- The used symbol referred to paragraph. To avoid any confusion, we have now replaced it by the word “paragraph”.

Comments from Reviewers:
Reviewer #2: The authors have addressed my concerns and I now recommend publication.
Peter Flom
-We thank Reviewer 2 again for his valuable comments.
Reviewer #3: Comments to the Author
Great job of responding to feedback. The manuscript still needs additional proofreading. Here are minor additional comments.
-We thank Reviewer 3 again for their positive inputs that helped us improve the quality of our paper.
Introduction  
Thanks for your explanation about obesity as a risk factor for metabolic disease.  I think it's valuable, though the writing needs also presents obesity as a metabolic disease (ICD-10 code E66).  The results can be maximized from that perspective. Presenting obesity as a disease is an important step in its management.  It is important to incorporate this discourse in our work on the topic.
-This has been added to the Introduction section, lines 104-105.
The first paragraph is long. I suggest that there are two paragraphs starting from line 110, which separates epidemiology from risk factors.
-This paragraph was now split to two as requested.
Line 115-121 - thanks for adding in this paragraph.  I think it's valuable, though the writing needs a more recent or a classic citation/reference for statement about food system and built environment. We suggest: (1) Swinburn BA, Kraak VI, Allender S, Atkins VJ, Baker PI, Bogard JR, et al. The Global Syndemic of Obesity, Undernutrition, and Climate Change: The Lancet Commission  report. The Lancet. 2019;393: 791-846. doi:10.1016/S0140-6736(18)32822-8; (2) Swinburn, B., Egger, G., Raza, F. Dissecting obesogenic environments: the development and application of a framework for identifying and prioritizing environmental interventions for obesity. Prev. Med., v.29, n.6, p.563-70, 1999.
-These references have now been cited.
Material and methods
Line 216-220 - insert the unit of measurement of the covariates: sugar, fibre, sodium, and saturated fatty acid (SFA), fruit, vegetables, and sugary drinks.
-Measurement units have been added.
Line 223-228 - why italics?
-Italics was used as this an Editorial requirement, that does not make part of our manuscript. We have now removed Italics.
Discussion
In the last paragraph, focus on France and exclude issues related to other countries like Brazil.
-Issues related to Brazil have been now excluded.

*****

All the production queries have been addressed.

In regards to data availability, data of the study are protected under the protection of health data regulation set by the French National Commission on Informatics and Liberty (Commission Nationale de l’Informatique et des Libertés, CNIL). The data can be available upon request to Nathalie Pecollo ([n.pecollo@eren.smbh.univ-paris13.fr](mailto:n.pecollo@eren.smbh.univ-paris13.fr)), after a consultation with the steering committee of the NutriNet-Santé study. The French law forbids us to provide free access to NutriNet-Santé data; access could be exceptionally given by the steering committee after legal verification of the use of the data. Please, feel free to come back to us should you have any additional question.
